# Living Together

**Published:** 1872-01

**Authors:** 


### LIVING TOGETHER.

A STRANGER has frequent opportunities of observing how families sometimes get into the habit of snapping at each other. Criticisms are incessant; and what is remarkable, they are almost always of the depreciative kind, seldom made with courtesy and kindness, but with an impatience and crustiness of tone which soon becomes simply ridiculous.

If a sister says, "We had a call at ten o'clock this morning," —

"It wasn't ten o'clock at all, it was only half past nine," starts up the eighteen-year-old brother.

We know of two old sisters, who have been living together about seventy years; one is all gentleness and yielding; the other is like a porcupine, darting off its quills at almost every other sentence. One says, —

"Last Wednesday morning we took a walk in the garden."

"Why, Arabella! you have no memory at all; it was after dinner."

And thus it goes, snap, snap, snap, all the day long.

Such a hypercritical spirit is always to be deprecated. It often induces rejoinders, contradictions, and angry discussions, which sometimes break up the enjoyment of a whole evening; it represses conversation, interferes with the freedom of social intercourse, and is everywhere a blot and a blur.

Such criticisms are contradictions, and contradictions are vulgar the world over. What we look for in the freedom of enjoyable conversation is the main fact of a statement. It is impossible for us to be always on the lookout — to be calculating the absolute correctness of every word and syllable uttered. It makes no possible difference whether a "clap of thunder" which killed the rooster on the fence, came half a second sooner or later. What if we do make a mistake of a day or a week, when we went to a "quilting party," or a "log rolling," or a "corn husking," or a "picnic;" it is not the exact day which is the point of the remark, but the meeting itself.

Away with this self-constituted censorship; it is an impertinence always.

The art of "living together" pleasurably is greatly promoted by the habitual exchange of the little courtesies of life; they are never unimportant, never unacceptable, are always grateful to the feelings, and are a constant wellspring of agreeable feelings in every household. Shall brothers and sisters be less careful of the feelings of one another, than those of a stranger? and as between husband and wife, should there be less effort at gentleness of deportment, of suavity of manner, and courtesy of expression than is extended to outsiders, who have no special claims and may never be seen again? Shame upon any member of any family who neglects those affectionate attentions, and those suavities of deportment towards the members of the household and even to the lowest servant, which cannot fail to elevate the giver, and to draw from the receiver those willing and spontaneous reciprocities which make of family associations a little heaven below.

### FAULT FINDING

Is an apple of discord in multitudes of families. There are some persons who, from ugliness of temper arising from bodily infirmity, or an inherent blight of nature, are forever finding fault, either with something said or done, or omitted to be said or done; if not in the family, then out of it. Somewhere or other something is always going wrong with them; in every remark they make there is vinegar and bitterness; their whole nature seems to be in a condition of chronic snarl; their adjectives are of the most sweeping character; everybody is a "liar" or "swindler" or "scoundrel," even if their shortcomings are of the slightest character. Such persons are demoralizers of the community in which they live, and of those with whom they associate; and as to the family in which they reside, they are a perpetual storm, a tornado, and a curse. This complaining, fault-finding trait does not assume these gigantic proportions of enormity at once, but always comes by slow degrees and long practice. Let the reader fear falling into this great condemnation; let him be so afraid of it from this good hour, as to resolve never to find fault with anybody or anything, or characterize any one's conduct for omissions or commissions, until he has "slept on it," thus giving the clearer judgment of a renovated brain an opportunity of more dispassionate exercise.

Let every person of intelligence, refinement, and culture, bear in mind that in "living together" with others pleasantly, happily, it is of essential importance to practice the virtues of uniform gentleness, deference, and courtesy, remembering that one of the most cardinal points in the promotion of domestic enjoyment and of family happiness is to cultivate self-sacrifice; for it is this which cherishes love in the heart of the giver, and kindles it in those for whom the self-sacrifice is made; or to frame the principle in a phrase which all can comprehend, remember and apply, That is the noblest heart in any household, which gives to the others the first choice, and leaves to others the best places and the best things.

